# Thermodynamic Insights by Microscale Thermophoresis into Translesion DNA Synthesis Catalyzed by DNA Polymerases Across a Lesion of Antitumor Platinum–Acridine Complex

**DOI:** 10.3390/ijms21207806

**Published:** 2020-10-21

**Authors:** Monika Hreusova, Olga Novakova, Viktor Brabec

**Affiliations:** 1 Czech Academy of Sciences, Institute of Biophysics, Kralovopolska 135, CZ-61265 Brno, Czech Republic; monca.hreusova@gmail.com (M.H.); olga@ibp.cz (O.N.); 2 Department of Biophysics, Faculty of Science, Palacky University, Slechtitelu 27, CZ 78371 Olomouc, Czech Republic

**Keywords:** platinum-acridine, antitumor, translesion DNA synthesis, DNA polymerases, microscale thermophoresis

## Abstract

Translesion synthesis (TLS) through DNA adducts of antitumor platinum complexes has been an interesting aspect of DNA synthesis in cells treated with these metal-based drugs because of its correlation to drug sensitivity. We utilized model systems employing a DNA lesion derived from a site-specific monofunctional adduct formed by antitumor [PtCl(en)(L)](NO_3_)_2_ (complex AMD, en = ethane-1,2-diamine, L = *N*-[2-(acridin-9-ylamino)ethyl]-*N*-methylpropionamidine) at a unique G residue. The catalytic efficiency of TLS DNA polymerases, which differ in their processivity and fidelity for the insertion of correct dCTP, with respect to the other incorrect nucleotides, opposite the adduct of AMD, was investigated. For a deeper understanding of the factors that control the bypass of the site-specific adducts of AMD catalyzed by DNA polymerases, we also used microscale thermophoresis (MST) to measure the thermodynamic changes associated with TLS across a single, site-specific adduct formed in DNA by AMD. The relative catalytic efficiency of the investigated DNA polymerases for the insertion of correct dCTP, with respect to the other incorrect nucleotides, opposite the AMD adduct, was reduced. Nevertheless, incorporation of the correct C opposite the G modified by AMD of the template strand was promoted by an increasing thermodynamic stability of the resulting duplex. The reduced relative efficiency of the investigated DNA polymerases may be a consequence of the DNA intercalation of the acridine moiety of AMD and the size of the adduct. The products of the bypass of this monofunctional lesion produced by AMD and DNA polymerases also resulted from the misincorporation of dNTPs opposite the platinated G residues. The MST analysis suggested that thermodynamic factors may contribute to the forces that governed enhanced incorporation of the incorrect dNTPs by DNA polymerases.

## 1. Introduction

Platinum-based coordination compounds offer broad possibilities for the design of anticancer chemotherapeutics [1]. The conventional platinum drugs, cisplatin, carboplatin, and oxaliplatin, are approved worldwide in the treatment of cancer. Currently, great effort has been devoted worldwide to the development of new, nonclassical platinum complexes that operate via mechanisms of action distinct from those of the approved platinum anticancer drugs [1,2,3,4,5]. Designing new antitumor platinum drugs and suggesting strategies for improving the chemotherapeutic effectiveness of the existing drugs depend on understanding the details of molecular and biochemical mechanisms associated with the biological effects of the existing agents. The antitumor activity of platinum complexes is connected with their ability to bind coordinatively to N7 atoms of purine bases in DNA in the nucleus of cancer cells forming intrastrand and interstrand crosslinks [2]. One of the principal effects of these DNA adducts is that they can stall DNA and RNA polymerases from synthesizing new strands of these nucleic acids [1,2,3]. This inhibition can trigger signal transduction pathways leading to cell death [4,5].

**Figure 1:**
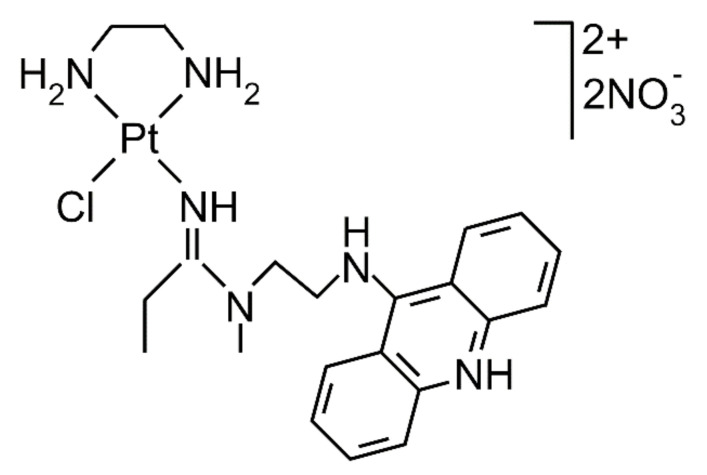
Structure of the investigated platinum complex AMD.

In efforts to identify new structural motifs in platinum complexes leading to antitumor effects superior to those of clinically used platinum drugs, such as cisplatin, carboplatin, or oxaliplatin, platinum–acridine hybrid antitumor compounds were synthesized and tested for their antiproliferative efficiency in cancer cells [6,7,8,9]. For instance, compound [PtCl(en)(L)](NO_3_)_2_ (complex ACR, en = ethane-1,2-diamine, L = 1-[2-(acridin-9-ylamino)ethyl]-1,3-dimethylthiourea) and an analog containing L = *N*-[2-(acridin-9-ylamino)ethyl]-*N*-methylpropionamidine (complex AMD) (see Figure 1 for its structure) have been shown to exhibit antiproliferative activity in several human cancer cell lines, including cell lines resistant to conventional cisplatin [10,11,12]. Additionally, the mechanism of biological action of these compounds is fundamentally different from that of conventional platinum drugs used in the clinic. Similarly to conventional anticancer platinum drugs, the main pharmacological target of platinum–acridine hybrid antitumor compounds is DNA, and it has been shown that the DNA-binding mode of ACR and AMD involves a combination of monofunctional coordination to purine residues and intercalation of the acridine moiety [13,14,15,16,17,18,19]. Interestingly, complex AMD was designed with the aim to modulate the cytotoxicity of ACR by replacing the thiourea donor group in the latter compound with an amidine group [11]. This modification resulted in enhanced formation of DNA adducts [11] and a more severe perturbation of the B-conformation of DNA. It was also shown [19] that DNA adduct geometries and the intercalation modes of ACR and AMD differ and that DNA adducts of AMD inhibit DNA replication more efficiently than those of ACR. Notably, although platinum–acridine hybrid agents do not form in DNA crosslinks, in contrast to conventional platinum antitumor drugs, their DNA adducts effectively inhibit DNA synthesis by DNA polymerases in treated cells and lead to stalled replication forks [7,20,21].

It has been shown that DNA adducts of antitumor platinum drugs arrest several key cellular functions, including DNA replication, and that this arrest triggers programmed cell death [3,4,5]. On the other hand, cells also try to recover halted DNA replication and rely on activities of translesion DNA polymerases (TLS pols) capable of bypassing the adducts. TLS pols are less strict than most replicative DNA polymerases and can more easily accommodate damaged bases and incorporate an incorrect nucleotide [22]. Thus, when DNA adducts of antitumor platinum drugs are formed, errors of DNA synthesis during replication may occur, inducing mutations. Another consequence of the action of TLS pols may consist of the reduced sensitivity of tumor cells to platinum drugs due to enhanced adduct tolerance mediated by an increased ability of TLS pols to replicate past platinum adducts.

There are several classes of DNA polymerases (DNA pols) that differ in specificity in their lesion-bypass properties, including bypass ability, fidelity, and extension ability [23,24,25]. The efficiency of DNA pols to insert correct nucleotides is affected not only at the site of the lesion, but also at positions several nucleotides away from the lesion, in particular in the 5′ direction [26,27,28,29,30]. The DNA lesions may also have long-range effects on DNA pol activity [31,32].

In the present work, we investigated the bypass of the site-specific monofunctional adduct of AMD catalyzed by TLS pols, which differ in their processivity and fidelity, by employing “running and standing start” assays [33]: Klenow fragment from DNA polymerase I (exonuclease minus, mutated to remove the 3′->5′ proofreading activity) (KF^−^) is a model and well-characterized enzyme frequently used in studies aimed at understanding the processes in which nucleic acid polymerases take part [34,35]; and DNA polymerase η (pol η) is a low fidelity TLS pol that rescues damage-stalled replication by inserting deoxyribonucleotides opposite DNA damage sites resulting in error-free or mutagenic damage bypass [36]).

Notably, the effects of the lesions formed in DNA by AMD on DNA pol activity are not only connected with structural confinements during the process of insertion of a correct or incorrect nucleotide. Still, they may also be a consequence of the energetic impact of the lesion on the process of translesion synthesis (TLS) [29,30,31]. Thus, for a deeper understanding of the factors that control the bypass of the site-specific adducts of AMD catalyzed by DNA pols, it is of considerable interest to assess also the thermodynamic data associated with DNA chain elongation [30,31]. In the present work, we also used microscale thermophoresis (MST) to measure the thermodynamic changes associated with translesion synthesis across single, site-specific adducts formed in DNA by AMD as well. The results of the studies presented in this report represent the first attempt to describe translesion DNA synthesis by DNA polymerases across the adduct of a platinum–acridine hybrid antitumor compound and the thermodynamic data associated with this process.

## 2. Results and Discussion

### 2.1. Sequence Specificity of the Formation of an AMD Adduct Formed in DNA by Transcription Mapping

We decided to carry out experiments demonstrating DNA polymerization by DNA polymerases and MST experiments with the DNA templates site-specifically modified by the single adducts of AMD. Therefore, it was necessary first to identify sites in DNA at which AMD preferentially forms the adducts. To accomplish this task, we used transcription mapping [37,38] of AMD-DNA adducts. The phenomenon that RNA synthesis by DNA-dependent RNA polymerases is prematurely terminated by the adducts of platinum compounds allowed us to identify preferential binding sites of the platinum complexes based on arrest sites of DNA transcription [39].

pSP73KB DNA (part of the nucleotide sequence of this plasmid used for mapping is shown in Supplementary Figure S1B) contained SP6 or T7 RNA polymerase promotors (in both strands close to their 3‘- ends (Supplementary Figure S1B)). The experiments were carried out using DNA globally modified by AMD at *r*_b_ = 0.02 or 0.01, for RNA synthesis by SP6 or T7 RNA polymerase, respectively, and cisplatin at *r*_b_ = 0.01 (Supplementary Figure S1A) (*r*_b_ is defined as the number of molecules of the platinum complex bound per nucleotide residue). RNA synthesis on the pSP73KB plasmid, modified by the monofunctional AMD and bifunctional cisplatin complexes, yielded fragments of defined sizes, which indicated that RNA synthesis on these templates was prematurely terminated. The major stop sites produced by AMD were mainly at guanine residues. For comparative purposes, the inhibition of RNA synthesis by DNA adducts of cisplatin is also shown (Supplementary Figure S1A) and demonstrates mostly identical termination sites like those for AMD. The sequence analysis revealed that the major bands resulting from the termination of RNA synthesis by the adducts of cisplatin and AMD preferentially appeared one or a half nucleotide preceding G sites and, to a considerably less extent preceding A sites (in AGAG or AGAGGA sequences). Collectively, AMD exhibited a base sequence selectivity similar to that of cisplatin. Nevertheless, the efficiency of the monofunctional adducts of AMD to terminate RNA synthesis was, in general, slightly reduced relative to that of cisplatin.

It has been shown that in vitro RNA synthesis by DNA-dependent RNA polymerases on a DNA template modified by several bifunctional Pt(II) compounds can be prematurely terminated at the level or in the proximity of the crosslinks [38,40]. On the other hand, monofunctional DNA adducts of some platinum complexes, such as [PtCl(dien)]^+^ (dien = diethylenetriamine = 1,4,7-triazaheptane) or [PtCl(NH_3_)_3_]^+^, are unable to terminate RNA synthesis [37,38]. Interestingly, in contrast, monofunctional AMD formed on DNA, the adducts that efficiently terminate RNA synthesis, thanks to its bulkier acridine ligand. AMD produced adducts in the sequences 5′-CTG, 5′-CGATG, and 5′-CGG. This finding is consistent with the observation that this derivative targets 5′-pyrimidine-guanine steps [11]. Additionally, unlike complex ACR, which also produces a high percentage of adenine adducts, the only platinum-containing DNA fragment isolated from the enzymatic digests of calf thymus (CT) DNA treated with AMD was 2′-deoxyguanosine [19]. Thus, the DNA templates (oligonucleotides) used in the experiments demonstrating DNA polymerization by DNA polymerases and MST experiments were site-specifically modified by the single adducts formed by AMD at G residues in the 5′-CG sequences.

### 2.2. Replication Through DNA Adducts of AMD by Purified TLS Pols

#### 2.2.1. Running-Start Primer Extension Experiments in the Presence of All Four Natural dNTPs

**Figure 2:**
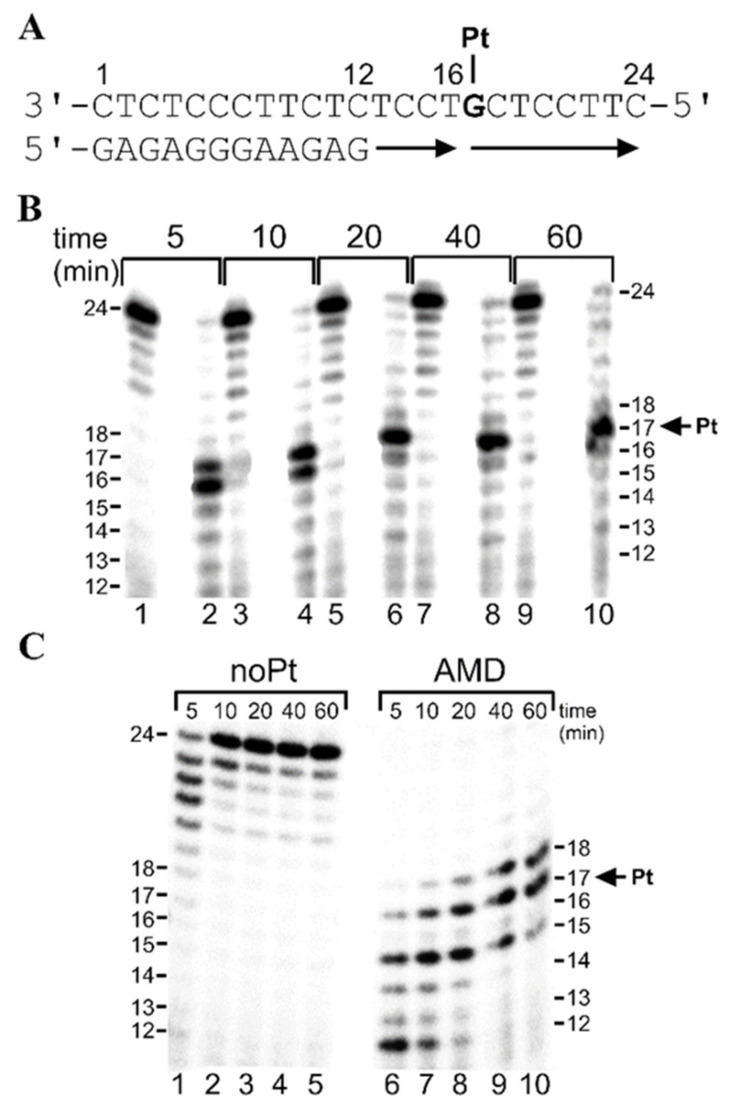
Translesion DNA synthesis by Klenow fragment (KF^−^) and DNA polymerase η (pol η) on the 12-mer/24-mer primer-template duplex non-modified or containing a single, monofunctional adduct of AMD in the presence of all four dNTPs (“running start” DNA synthesis) for various time intervals (time points of 5–60 min are shown above the gels). The sequence of the primer–template sequence is shown in (**A**) and the position of the platinated G residue is indicated. Representative images of the DNA polymerase reaction products resolved on 15% polyacrylamide (PAA) gel are shown for DNA synthesis (**B**) by KF^−^ in the presence of 25 μM of each of the four dNTPs and (**C**) by pol η in the presence of 100 μM of each of the four dNTPs. Lanes in Figure 2B: 1, 3, 5, 7, 9, undamaged template; 2, 4, 6, 8, 10, the template containing the single, site-specific monofunctional adduct of AMD. Lanes in (**C**): 1–5, noPt, DNA synthesis using an undamaged template; 6–10, AMD, DNA synthesis using the template containing the single, monofunctional adduct of AMD. The pause sites (the product lengths) are shown on the right or left side of the gels.

It has been demonstrated that DNA modifications by various DNA-damaging agents have significant effects on the processivity of several prokaryotic, eukaryotic, and viral DNA polymerases [41,42,43,44,45,46,47]. Interestingly, with DNA templates containing site-specifically placed adducts of various DNA-damaging compounds, a number of prokaryotic and eukaryotic DNA polymerases were blocked, but could also traverse through platinum adducts, depending on their character and conformational alterations induced in DNA. In the present work, we investigated DNA polymerization using the template that was site-specifically modified by the adduct of AMD by KF^−^ and pol η. We constructed, for the first series of experiments (“running start” [48]), 12-mer/24-mer primer-template duplexes (Figure 2A) that were non-modified or contained a single adduct of AMD.

The first 12 nucleotides on the 3’ terminus of the 24-mer template strand were complementary to the nucleotides of the 12-mer primer, so that the 3’ guanine at which the adduct of AMD was formed on the template strand was located at its 17th position from the 3’ terminus (Figure 2A). After annealing the 12-nucleotide primer to the 3’ terminus of the non-modified or adduct-containing template strand (positioning the 3’ end of the primer five bases before the adduct in the template strand), we examined DNA polymerization through the adduct of AMD by KF^−^ and pol η in the presence of all four dNTPs. The reaction was stopped at various time intervals, and the products were analyzed using a sequencing gel (Figure 2B,C). Polymerization by KF^−^ using the 12-mer/24-mer primer-templates containing the adduct of AMD in the presence of all four dNTPs proceeded rapidly up to the nucleotide opposite the base immediately downstream from the adduct and the 3’ guanine involved in the adduct, such that the 16- and 17-nucleotide intermediate products accumulated to a significant extent (Figure 2B). The polymerization produced markedly more 17-nucleotide intermediate products, in particular, at longer reaction times. There was only a slight accumulation of longer DNA intermediates or full-length (24-nucleotide) products. Mainly full-length products were seen with the 24-mer non-modified control template (Figure 2B). Conclusively, the AMD adduct was very potent in inhibiting TLS activity of the high-fidelity DNA polymerase KF^−.^

Polymerization by a prototypical TLS polymerase pol η using the 12-mer/24-mer primer–templates containing the adduct of AMD in the presence of all four dNTPs proceeded differently (Figure 2C). The polymerization proceeded rapidly up to the nucleotide opposite the base two bases downstream from the adduct and the 3’ guanine involved in the adduct, such that the 15–18-nucleotide intermediate products accumulated to a significant extent. Interestingly, the adduct of AMD efficiently terminated DNA synthesis already at the nucleotide opposite the base five bases downstream from the adduct at shorter reaction times (5–10 min). Moreover, there was only a very slight accumulation of DNA intermediates longer than 18 nucleotides or full-length (24-nucleotide) products.

#### 2.2.2. Standing-Start Primer Extension Experiments in the Presence of All Four Natural dNTPs

**Figure 3:**
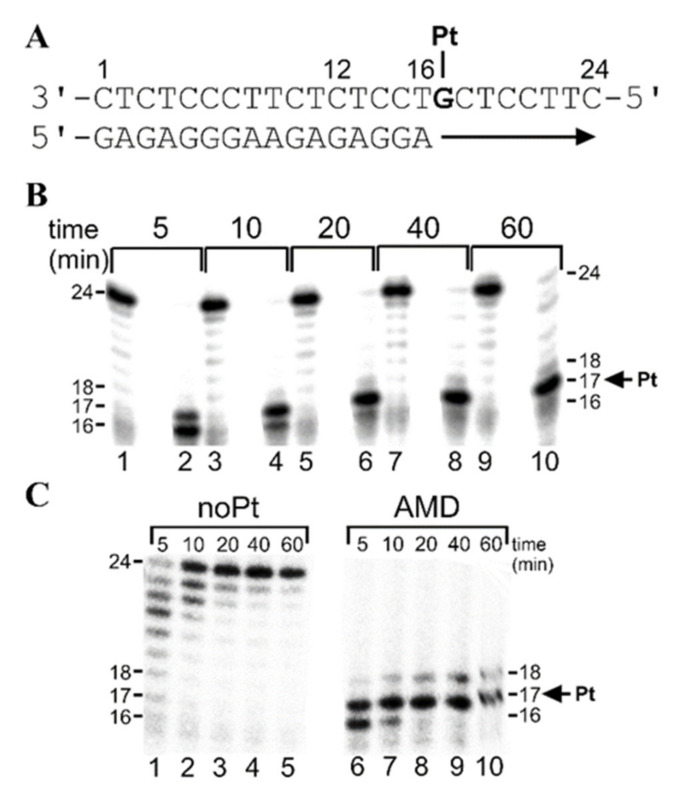
Translesion DNA synthesis by KF^−^ and pol η on the 16-mer/24-mer primer–template duplex non-modified or containing a single, monofunctional adduct of AMD in the presence of all four dNTPs (“standing start” DNA synthesis) for various time intervals (time points of 5–60 min are shown above the gels). The sequence of the primer–template is shown in (**A**); the position of the platinated G residue is indicated. Representative images of the DNA polymerase reaction products resolved on 15% polyacrylamide (PAA) gel are shown in (**B**) for DNA synthesis by KF^−^ in the presence of 25 μM of each of the four dNTPs and in (**C**) for DNA synthesis by pol η in the presence of 100 μM of each of the four dNTPs. Lanes shown in (**B**): 1, 3, 5, 7, 9, undamaged template; 2, 4, 6, 8, 10, the template containing the single, site-specific monofunctional adduct of AMD. Lanes shown in (**B**): 1–5, noPt, DNA synthesis using an undamaged template; 6–10, AMD, DNA synthesis using the template containing single, monofunctional adduct of AMD. The pause sites (the product lengths) are shown on the right or left side of the gels.

For the other series of experiments (“standing start” [48]), we constructed 16-mer/24-mer primer–template duplexes (Figure 3A) that were non-modified or contained a single adduct of AMD. The first 16 nucleotides on the 3’ terminus of the 24-mer template strand were complementary to the nucleotides of the 16-mer primer (Figure 3A). Thus, after annealing the 16-nucleotide primer to the 3’ terminus of the non-modified or adduct-containing template (positioning the 3’ end of the primer only one base before the adduct in the template strand), we examined DNA polymerization through the adduct of AMD by KF^−^ and pol η in the presence of all four dNTPs in the same way as in the case of the “running start” experiments (Figure 2). Polymerization by both investigated polymerases proceeded qualitatively similarly as in the case of the “running start” experiments. The only noticeable difference consisted of less extensive accumulation of longer DNA intermediates or full-length (24-nucleotide) products. In aggregate, in the presence of all four dNTPs, pol η exhibited higher bypass efficiency past the AMD adduct compared with KF^−^.

#### 2.2.3. Steady-State Kinetics of dNTP Incorporation by KF^−^ and Pol η Opposite G Unplatinated or Platinated by AMD

For a more quantitative characterization of nucleotide incorporation opposite the G sites platinated by AMD by the two representative DNA polymerases KF- and pol η, kinetic parameters for nucleotide insertion and extension during TLS were measured on templates containing a single, site-specific, monofunctional adduct of AMD and on undamaged templates using the 16-mer/24-mer primer–template duplex. The kinetics of the insertion of a single deoxyribonucleotide, opposite the platinated guanine to the 3′-primer termini situated across from the platinated guanines, were determined as a function of the deoxyribonucleotide concentration under steady-state conditions. For comparison, the kinetic parameters for the insertion opposite an unplatinated template G were measured as well. From the kinetics of the deoxyribonucleotide incorporation, the steady-state apparent *K*_m_ and *V*_max_ values for each incoming deoxyribonucleotide opposite the template G platinated by AMD, or opposite the same G in the unplatinated template, were obtained from curve fitting by the Michaelis–Menten equation as described in Section 4.6. The patterns and plots of dCTP, dATP, dGTP, or dTTP incorporation by KF^−^ and pol η opposite the G residue platinated by AMD are shown in Supplementary Figures S2–S4. The *K*_m_ and *V*_max_ parameters were determined and used to calculate the percentage of each nucleotide incorporated opposite the platinated G residues (Table 1 and Table 2).

The values of *V*_max_/*K*_m_ showed that dCMP was most prevalently inserted against the AMD adduct by both DNA polymerases. Interestingly, the relative efficiency of KF^−^ to incorporate the correct dCTP opposite the G residue platinated by AMD was only 0.02 (Table 1). The relative efficiency of the insertion of other (incorrect) nucleotides was 0.11 for dATP, 0.06 for dGTP, and 0.01 for dTTP. Thus, this analysis indicated that KF^−^ incorporated the correct dCTP opposite the platinated G less than other incorrect nucleotides dATP and dGTP with a 5- and 3-fold higher relative efficiency than the correct dCTP, respectively.

Next, we examined the relative efficiency of low fidelity pol η to incorporate the correct or incorrect nucleotides opposite the G residue platinated by AMD. The *K*_m_ and *V*_max_ parameters were obtained and used to calculate the relative efficiency for incorporating each nucleotide similarly as described in the preceding paragraph (Table 2). The relative efficiency of pol η to incorporate the correct dCTP opposite the G platinated by AMD was only slightly higher (1.5-fold) than that of KF^−^. The relative efficiency for the insertion of incorrect nucleotides was markedly higher than that of KF^−^; the relative efficiency for the insertion of incorrect dATP, dGTP, and dTTP was 0.43, 1.14, and 0.55, respectively. Thus, this analysis indicated that pol η incorporated correct dCTP opposite the G platinated by AMD considerably less than the other incorrect nucleotides, although pol η was apparently the more error-prone polymerase than KF^−^. Additionally, the highest relative efficiency of pol η to incorporate incorrect (mismatched) dNTP opposite the G platinated by AMD was observed for the incorporation of dGTP.

### 2.3. Probing the Thermodynamics of Translesion DNA Synthesis Across a Monofunctional Adduct of AMD by Microscale Thermophoresis (MST)

**Figure 4:**
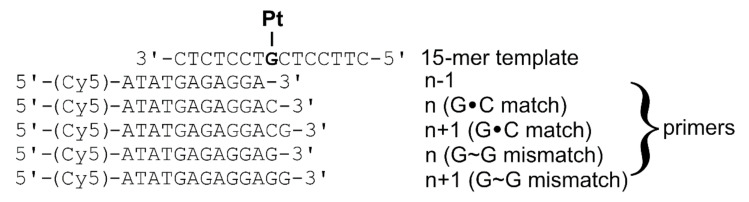
Sequences of 15-mer template and primers n − 1, n, and n + 1. The bold letter G in the template indicates the AMD adduct.

Here, we show (Table 1 and Table 2) that the monofunctional adduct of AMD considerably affects the insertion of the correct nucleotide opposite this adduct formed at the G residue by KF^−^ and pol η. It was suggested [31,49] that, for a deeper understanding of the nature and magnitude of the forces that control the mechanisms of polymerase-mediated recognition and catalysis associated with template-directed DNA synthesis, it is important to know the thermodynamic data associated with DNA chain elongation. In the present work, we used MST to measure the thermodynamic changes associated with translesion synthesis across a monofunctional adduct formed by AMD at the G residue. Two template–primers were designed to simulate translesion synthesis across this monofunctional adduct. The probe, designated “15-mer template” in Figure 4 and containing the unique G residue, which was either unplatinated or modified by the monofunctional adduct of AMD, was paired with two sets of primers to simulate TLS across the AMD adduct. The probe (15-mer template) was paired with the match and mismatch series (Figure 4). G residue was chosen for the mismatched series because among incorrect (mismatched) dNTPs, dGTP was incorporated by KF^−^ and pol η with a relatively high efficiency (Table 2 and Table 3). Interestingly, pol η incorporated the incorrect dGTP opposite the platinated G residue even with higher efficiency than opposite the G residue in the non-modified control template. Thus, the probe (15-mer template) was hybridized with the oligonucleotides designated as primers n − 1, n (G∙C match), n + 1 (G∙C match), n (G~G mismatch), and n + 1 (G~G mismatch). The primers n − 1, n, and n + 1 were chosen for these studies because the most pronounced structural perturbations induced in double-helical DNA by AMD adducts were also observed at base pairs adjacent to the base-pair containing the AMD adduct [19].

The MST measurements required to use Cy5-labeled complementary primers so that the primers were prolonged on their 5′ sites by the sequence of four nucleotides (5′-ATAT) to eliminate the possible effects of Cy5 fluorophore on hybridized DNA duplexes. The MST experiments were performed in a medium of 10 mM phosphate buffer, pH 7, 150 mM NaCl, and 0.05% TWEEN, as described in the experimental part and Supplementary Figure S5.

In quantifying the energetics of the interactions of the DNA templates and primers, the equilibrium dissociation constants (*K*_d_) were determined for temperatures in the temperature range of 295–313 K, as described in the previously published study [50] using the software provided by the manufacturer for the evaluation of MST experiments [51]. The results are listed in Table 3. All thermodynamic parameters discussed in this work refer to the duplex dissociation process.

The thermodynamic data summarized in Table 3 discloses several noteworthy features. The insertion of a correct (matched) C base at the primer n − 1 terminus markedly increased the enthalpy of dissociation of duplexes 15-mer template/primer n or 15-mer template/primer n + 1, either unplatinated or containing the AMD adduct. In other words, the insertion of the correct C base at this primer terminus caused an enthalpic stabilization of the duplexes relative to their 15-mer template/primer n − 1 counterparts. On the other hand, the insertion of the correct C base at the primer n − 1 terminus resulted in a substantial increase in the dissociation entropy of the duplexes unplatinated or containing the AMD adduct (Table 3). Thus, the net result of these enthalpic and entropic effects was that the insertion of the matched C base induced an increase in the free energy of the duplex dissociation at 310 K (Δ*G*^0^_310_) (Table 3), a duplex stabilization being enthalpic in origin. Interestingly, a similar entropic compensation effect was also observed in the 15-mer template/primer n + 1 duplex. This observation suggested that the incorporation of C opposite the AMD lesion showed a change in the melting thermodynamics. The impact of the AMD lesion was also pronounced if the duplex was further prolonged 5′ downstream behind the AMD lesion. Nevertheless, the data showed that the enthalpy terms were primarily responsible for the adduct-induced thermodynamic stabilization.

Similar experiments were also performed for the G-mismatch series (Figure 4, Table 3). Unlike the C-match cases described above, the insertion of a mismatched G base at the terminus of primer n-1 differently affected the thermodynamic stability of the unplatinated and AMD-modified 15-mer template/primer n or 15-mer template/primer n + 1 duplexes (Δ*H* and Δ*S* were increased much less, whereas Δ*G*^0^_310_ was even slightly decreased (Table 3)). The data also showed that entropy terms were primarily responsible for this AMD adduct-induced duplex destabilization, thus being entropic in origin.

The Δ*G*^0^_310_ value observed for the melting of the 15-mer template/primer n (G∙C match) or 15-mer template/primer n + 1 (G∙C match) (i.e., the primer–template duplexes (Figure 4) positioning the correct C base at the 3′ end of the primer opposite the G in the template strand) was increased by 5.3 or 5.1 kJmol^−1^ as a consequence of the presence of the monofunctional adduct of AMD, respectively. Interestingly, this change in Δ*G*^0^_310_ represented an equilibrium preference for the duplexes modified by AMD over those which were unmodified. Thus, this result supported the view that the AMD monofunctional adduct is responsible for the marked thermodynamic stabilization of the DNA, which is of entropic origin.

It has been proposed that the effect of enthalpy–entropy compensation is suppressed in the enzyme catalytic pocket so that the insertion preference is primarily modulated by the enthalpy term [52]. The results shown in Table 3 indicated that not only the incorporation of correct dCMP, as well as incorrect dGMP, opposite the AMD adduct, was enthalpically favorable, but also that the incorporation of incorrect dGMP was markedly less enthalpically favorable than the incorporation of matched dCMP. This finding was in good agreement with experimental data (Figure 2 and Figure 3) showing that the AMD adduct blocked DNA polymerases one or more nucleotides prior to the lesion; if bypassed, dCMP was preferred over dGMP (Table 2).

Intercalation of the acridine moiety into the 5’ base pair step adjacent to the G residue platinated by AMD and associated delocalized conformational distortions [19] were also consistent with the observation that the AMD adduct had a pronounced deleterious effect on the energetics of the 15-mer template/primer n or n + 1 (G∙C match) duplexes (the transition enthalpy of these duplexes was considerably decreased by the AMD adduct) (Table 3). Taken together, our results are consistent with the view that the considerably reduced relative efficiency of KF^−^ and pol η to incorporate dCTP at the G residues platinated by AMD may be a consequence, at least partially, of the DNA intercalation of the acridine moiety into the 5’ base-pair step adjacent to the G residue platinated by AMD and accompanying delocalized conformational distortions [19].

The size of the adduct could also contribute to the blocking of KF^−^ and pol η and to the loss of fidelity for the incorporation opposite to the AMD adduct [53]. The DNA-binding mode of AMD involves a combination of intercalation and monofunctional coordination preferentially with the N7 donor site of G residues [19]. Thus, the AMD adducts reside in the major groove so that their direct interaction with KF^−^ and pol η is unlikely. Nevertheless, the AMD adducts could influence the strength of the DNA polymerase-DNA interaction if, for instance, the adduct hinders the additional conformational alterations of the DNA required to form the DNA polymerase-DNA incoming dNTP complex.

The Δ*G*^0^_310_ value observed for the melting of the 15-mer template/primer n − 1 duplex (that is, the duplex positioning the complementary nucleotide at the 3′ end of the primer only one base before the G site in the template strand so that no base was opposite the G base involved in the adduct (Figure 4)) was increased by 7.2 kJmol^−1^ as a consequence of the presence of the AMD adduct. This result could be interpreted to mean that the AMD adduct increased the thermodynamic stability of the base pairs in the close proximity of the adduct also on its 3′ site. This interpretation is consistent with the previous observation that the conformational distortion induced by the AMD adduct is delocalized and can also affect base pairs in the immediate neighborhood of the AMD adduct [19].

## 3. Conclusions

We investigated DNA polymerization using the template site-specifically modified by the adduct of antitumor AMD formed at the guanine (G) residue by KF^−^ and pol η in the presence of all four dNTPs. The results indicated that pol η exhibited higher bypass efficiency past the AMD adduct compared with KF^−^ and that dCMP was most prevalently inserted against the modified guanine residue by both DNA polymerases. Pol η incorporated correct dCTP opposite the G platinated by AMD considerably less than the other incorrect nucleotides, although pol η was apparently the more error-prone polymerase than KF^−^. Additionally, the highest relative efficiency of pol η to incorporate incorrect (mismatched) dNTP opposite the G platinated by AMD was observed for the incorporation of dGTP. For a deeper understanding of the nature and magnitude of the forces that control the mechanisms of polymerase-mediated recognition and catalysis associated with template-directed DNA synthesis, we also determined the thermodynamic changes associated with translesion synthesis across the monofunctional adduct formed by AMD at the G residue. The thermodynamic data disclosed several noteworthy features. The insertion of the matched C base induced an increase in the free energy of the duplex dissociation at 310 K (Δ*G*^0^_310_), a duplex stabilization being enthalpic in origin. This result supported the view that the AMD monofunctional adduct is responsible for the marked thermodynamic stabilization of the DNA, which is of entropic origin. The thermodynamic data for the incorporation of incorrect dGMP opposite the AMD adduct were measured as well and indicated that this incorporation was markedly less enthalpically favorable than the incorporation of matched dCMP. This finding was in good agreement with experimental data showing that the AMD adduct blocked DNA polymerases one or more nucleotides prior to the lesion; if bypassed, dCMP was preferred over dGMP.

In aggregate, the results demonstrated that incorporation of the correct C opposite the G modified by AMD was promoted by an increasing thermodynamic stability of the resulting duplex. This can be expected because the C residue is, in this case, a part of the Watson-Crick duplex so that the addition of a further complementary base pair increases the thermodynamic stability of the duplex. On the other hand, the incorporation of incorrect dGTP does not increase the thermodynamic stability of the duplex, because G is unable to form a Watson–Crick base pair with the G in the double-helical DNA. In agreement with this assumption, promiscuous insertion of dGTP even decreased the thermodynamic stability of the G~G mismatch duplex, either of the unplatinated one or the one containing the AMD adduct. Therefore, our results support the thesis that one of the reasons why KF^−^ and pol η incorporated incorrect dGTP opposite the G residues modified by AMD more efficiently may also be a little effect of this misincorporation on the thermodynamic stability of the duplex.

In summary, this study demonstrated the usefulness of MST for evaluating the detailed thermodynamics of translesion DNA synthesis across the adduct of antitumor AMD. The MST results were in good agreement with experimental data describing translesion DNA synthesis across the AMD adduct by DNA polymerases of eukaryotic and prokaryotic origin. The equilibrium thermodynamic data also contributed to a deeper understanding of the factors that control the mechanisms of template-directed DNA synthesis across the AMD adduct, and provided insight into the thermodynamic contributions to the insertion of dNTPs opposite the AMD adduct by DNA polymerases.

## 4. Materials and Methods

### 4.1. Chemicals

Riboprobe System-SP6/T7 for transcription mapping containing T7 and SP6 RNA polymerases was purchased from Promega (Madison, WI, USA), and the pSP73KB (2455 bp) plasmid was isolated according to standard procedures. Cisplatin was obtained from Sigma-Aldrich s.r.o. (Prague, Czech Republic). The synthetic oligodeoxyribonucleotides and Cy5-labeled DNA primers were purchased from Eurofins Genomics (Ebersberg, Germany). Full-length human DNA polymerase η (XPV protein) was purchased from EnzyMax LLC (Lexington, KY, USA). The exonuclease deficient Klenow fragment (KF^−^), T4 polynucleotide kinase, and dNTPs were purchased from New England Biolabs (Beverly, MA, USA). Acrylamide, bis(acrylamide), and urea were from Merck KgaA (Darmstadt, Germany). Radioactive products were from M.G.P. (Zlin, Czech Republic).

### 4.2. Transcription Mapping of DNA Platinum Adducts

Transcription of the pSP73KB plasmid DNA with SP6 or T7 RNA polymerase and electrophoretic analysis of transcripts were performed according to the protocols recommended by Promega (Promega Protocols and Applications, 43-46 (1989/90)) and previously described in detail [38]. Plasmid DNA was incubated with AMD or cisplatin in 0.1 × TE buffer at 310 K for 24 h in the dark. The number of molecules of the platinum compound coordinated per nucleotide residue (*r*_b_ values) was determined by GF AAS and EAS (spectrophotometrically). The concentration of DNA used in this assay was 7.8 × 10^−5^ M (0.5 µg/20 µL) (relative to the monomeric nucleotide content).

### 4.3. Platination of Oligonucleotides

The oligonucleotides 24-mer 5′-CTTCCTC**G**TCCTCTCTTCCCTCTC-3′ and 15-mer 5′-CTTCCTC**G**TCCTCTC-3′ were allowed to react with platinum complex AMD in a 1:1 molar ratio, and then the platinated oligonucleotides were purified by anion-exchange HPLC. It was verified by flameless atomic absorption spectrometry (FAAS) and by the measurements of the optical density that the modified oligonucleotides contained one platinum atom. It was also verified using Maxam–Gilbert DMS footprinting [38,54] that one molecule of AMD platinum complex was coordinated to the N7 atom of the G in each strand of these template oligonucleotides. HPLC purification and FAAS measurements were carried out on a Waters 600S Controller HPLC system with MonoQ HR 5/5 column and a Varian AA240Z Zeeman atomic absorption spectrometer equipped with a graphite tube atomizer (GTA 120), respectively.

### 4.4. Translesion Synthesis Assays

The primer extension assays with all four dNTPs were performed with the 24-mer template containing a single monofunctional adduct of AMD platinum compound, which was prepared as described above, and an unplatinated template.

The 5′-^32^P-labeled primer–template DNA substrate was obtained by mixing a 12-mer 5′-GAGAGGGAAGAG-3′ or 16-mer 5′-GAGAGGGAAGAGAGGA-3′ primer (radiolabeled at its 5′ end) with the 24-mer template 5′-CTTCCTC**G**TCCTCTCTTCCCTCTC-3′ at a molar ratio of 1:3 in 20 mM NaClO_4_, followed by hybridization for 10 min at 328 K and 2 h at room temperature.

All experiments using KF^−^ were performed at 298 K in 25 μL buffer containing 50 mM NaCl, 10 mM Tris-HCl (pH 7.9), 10 mM MgCl_2_, 1 mM DTT, 100 μgmL^−1^ BSA, 40 nM of the 5′-^32^*P*-labeled primer–template, 0.5 U of KF^−^, and the four dNTPs at 25 μM each.

All experiments using polymerase η were performed at 310 K in 25 µL buffer containing 40 mM Tris-HCl (pH 8.0), 2 mM MgCl_2_, 10 mM DTT, 250 μgmL^-1^ BSA, 60 mM KCl, 2.5% glycerol, 40 nM of the 5′-^32^*P*-labeled primer–template, pol η (1 ngμL^−1^), and the four dNTPs at 100 µM each.

At appropriate time intervals (5, 10, 20, 40, and 60 min), sample aliquots (5 µL) were withdrawn, and all enzymatic reactions were terminated by the addition of 2 µL of stop solution containing 95% formamide, 20 mM EDTA, 0.025% bromophenol blue, and 0.025% xylene cyanol. The products were denatured by boiling at 363 K for 3 min and separated by electrophoresis on a denaturing 15% PAA gel. Gels were visualized using the Typhoon FLA 7000 bioimaging analyzer and analyzed using the AIDA bio-image analyzer software (Raytest, Straubenhardt, Germany).

### 4.5. Nucleotide Misinsertion by KF^−^ and Human Polymerase η

Experiments were performed under the same reaction conditions as translesion synthesis assay studies of individual polymerases in the steady-state (60 min) in the presence of all four deoxyribonucleotide 5‘-triphosphates or selected dNTPs, complementary dCTP, or non-complementary dATP, dGTP, and dTTP (100 µM each). Reactions were terminated, as described above.

### 4.6. Steady-State Kinetic Analysis for dNTP Incorporations by KF^−^ and Pol η 

A steady-state kinetic analysis for dNTP incorporation opposite the unplatinated or platinated G (in the template, 3’ oligonucleotide with the monofunctional adduct of AMD) catalyzed by KF^−^ and human polymerase η was done as described previously [48,55,56,57]. The same amount of polymerase η and KF^−^, under the same reaction conditions as in nucleotide fidelity experiments mentioned above, was incubated with hybridized 16-mer primer–template in the presence of individual dNTPs (increasing concentration 0.125–1000 µM of examined dNTP) for 10 min. Reactions were terminated as in previous experiments. Gel band intensities of the substrates and products were visualized and quantified. The percentage of 16-mer primer extension (the sum of the intensities of bands corresponding to all products 17 and/or extended 17+ [48,55,56,57]) was plotted as a function of product concentration, and the data were fitted by non-linear regression using GraphPad software to the Michaelis–Menten equation describing hyperbola, *v* = *V*_max_ × [dNTP]/*K*_m_ + [dNTP].

Apparent *K*_m_ and *V*_max_ steady-state parameters were obtained from the best fit and were used to calculate the relative efficiency (RE) of the dNTP insertion opposite the template:

RE = (*V*_max_/*K*_m_)_platinated template_/(*V*_max_/*K*_m_)_unplatinated template_.

### 4.7. Microscale Thermophoresis (MST)

MST is a suitable technique for obtaining the thermodynamic parameters of modified oligonucleotides [50]. We calculated the DNA hybridization equilibrium binding constant *K*_a_ of a 15-mer oligonucleotide and a Cy5-labeled complementary primer over a range of temperatures (295–313 K) using a Monolith NT115 Pico instrument. DNA hybridization can be readily detected by MST due to the different thermophoretic signals of ssDNA and dsDNA. Serial dilutions of platinated 15-mer 5′-CTTCCTC**G**TCCTCTC-3′ and unplatinated templates (5 × 10^−6^ M–3 × 10^−11^ M) were paired with a set of 5’-Cy5-labeled primers (from n - 1 to n + 1; 5′-ATATGAGAGGA-3′, 5′-ATATGAGAGGAC-3′, 5′-ATATGAGAGGACG-3′, and 5’-ATATGAGAGGACGA-3′; 1–4 nM) in the volume ratio 1:1 in 10 mM phosphate buffer (pH 7.0), 150 Mm NaCl, and 0.05% TWEEN.

All the reaction solutions were filled into the “standard” or “premium” glass capillaries and, after stabilizing the required temperature, immediately measured by the MST instrument. Data analyses and curve fitting were carried out using the Nanotemper Analysis software. All the experimental parameters used by the MST instrument were fixed with a LED power of 20–100%, and a laser power of 40%. *K*_d_ values for each temperature were calculated and plotted in a van’t Hoff plot as ln(*K*_a_) vs. 1/T. The temperature dependence of the association constant *K*_a_ (which equals 1/*K*_d_) was used to deduce the thermodynamic parameters Δ*H* and Δ*S* by linear extrapolation of the data in the van’t Hoff plot. Δ*G* was calculated by using equation Δ*G* = Δ*H* – *T*Δ*S*. Thermodynamic parameters of platinated oligonucleotide were compared with unplatinated control.

## Figures and Tables

**Table 1:** Kinetics of Incorporation by KF^−^ of dNTPs opposite the G Platinated by AMD.

DNA Substrate	dNTP	*K*_m_ (μM)	*V*_max_ (%/min)	*V*_max_/*K*_m_	RE ^1^
5′----A 3′----TGCT----	dCTP	0.033 ± 0.003	9.88 ± 0.06	299.3	
	dATP	2.4 ± 0.7	0.58 ± 0.05	0.242	
	dGTP	5 ± 1	10.5 ± 0.9	2.1	
	dTTP	5.4 ± 0.7	12.2 ± 0.6	2.259	
5′----A 3′----TGCT---- • AMD	dCTP	1.5 ± 0.8	8 ± 1	5.333	0.02
	dATP	15 ± 2	0.4 ± 0.1	0.027	0.11
	dGTP	4 ± 1	0.47 ± 0.07	0.118	0.06
	dTTP	10 ± 2	0.33 ± 0.09	0.033	0.01

^1^ RE (relative efficiency) compares the efficiency (*V*_max_/*K*_m_) of the particular dNTP insertion opposite the template platinated G to the efficiency of the same dNTP insertion opposite the G, which was unplatinated.

**Table 2:** Kinetics of Incorporation by Pol η of dNTPs opposite the G Platinated by AMD.

DNA substrate	dNTP	*K*_m_ (µM)	*V*_max_ (%/min)	*V*_max_/*K*_m_	RE ^1^
5′----A 3′----TGCT----	dCTP	0.8 ± 0.2	9.9 ± 0.6	12.36	
	dATP	450 ± 20	3.0 ± 0.8	0.007	
	dGTP	78 ± 4	2.8 ± 0.6	0.036	
	dTTP	93 ± 7	7.8 ± 0.8	0.084	
5′----A 3′----TGCT---- • AMD	dCTP	18 ± 4	7.4 ± 0.6	0.411	0.03
	dATP	409 ± 24	1.1 ± 0.3	0.003	0.43
	dGTP	68 ± 21	2.8 ± 0.8	0.041	1.14
	dTTP	124 ± 12	5.7 ± 0.8	0.046	0.55

^1^ RE (relative efficiency) compares the efficiency (*V*_max_/*K*_m_) of the particular dNTP insertion opposite the template platinated G to the efficiency of the same dNTP insertion opposite the G, which was unplatinated.

**Table 3:** Microscale thermophoresis (MST)-derived thermodynamic parameters for the dissociation (melting) of the duplexes: 15-mer template/primer n − 1, 15-mer template/primer n (G∙C match), 15-mer template/primer n + 1 (G∙C match), 15-mer template/primer n (G~G mismatch), and 15-mer template/primer n + 1 (G~G mismatch), unmodified or containing a single, site-specific adduct of AMD formed at the G residue.

15-mer Template/ Primer n − 1 ^1^	Δ*H^b^* (kJmol^−1^)	Δ*S*^2^ (kJK^−1^mol^−1^)	Δ*G*^0^_310_^2^ (kJmol^−1^)	*K*_d_^3^ (nM)
noPt (control)	187	0.511	28.5	15,846
AMD adduct	225	0.612	35.2	1179
15-mer template/ primer n (G∙C match)^1^	Δ*H^b^* (kJmol^−1^)	Δ*S*^2^ (kJK^−1^mol^−1^)	Δ*G*^0^_310_^2^ (kJmol^−1^)	*K*_d_^3^ (nM)
noPt (control)	295 (108)	0.853 (0.342)	30.4 (1.9)	7585
AMD adduct	259 (34)	0.720 (0.108)	35.7 (0.5)	971
15-mer template/ primer n + 1 (G∙C match)^1^	Δ*H^b^* (kJmol^−1^)	Δ*S*^2^ (kJK^−1^mol^−1^)	Δ*G*^0^_310_^2^ (kJmol^−1^)	*K*_d_^3^ (nM)
noPt (control)	327 (140)	0.935 (0.424)	37.0 (8.5)	587
AMD adduct	293 (68)	0.809 (0.197)	42.1 (6.9)	81
15-mer template/ primer n (G~G mismatch)^1^	Δ*H^b^* (kJmol^−1^)	Δ*S*^2*b*^ (kJK^−1^mol^−1^)	Δ*G*^0^_310_^2^ (kJmol^−1^)	*K*_d_^3^ (nM)
noPt (control)	204 (17)	0.573 (0.062)	26.3 (−2.2)	37,193
AMD adduct	250 (25)	0.715(0.103)	28.2 (−7.0)	17,802
15-mer template/ primer n + 1 (G~G mismatch)^1^	Δ*H^b^* (kJ mol^−1^)	Δ*S*^2^ (kJK^−1^mol^−1^)	Δ*G*^0^_310_^2^ (kJmol^−1^)	*K*_d_^3^ (nM)
noPt (control)	245 (58)	0.700 (0.189)	27.9 (−0.6)	19,998
AMD adduct	267 (42)	0.766 (0.154)	29.4 (−5.8)	11,178

^1^ The nucleotide sequences of these duplexes are shown in Figure 4. ^2^ The ∆*H* and ∆*S* values are averages derived from two independent experiments. The experimental uncertainties of the parameters are as follows: ∆*H*, ±3%; ∆*S*, ±5%; ∆*G*^0^_310_, ±1%; *K*_d_, ±10%. “∆∆” parameters (in parentheses) are computed by subtracting the appropriate value measured for the 15-mer template/primer n − 1, from the value measured for the duplex 15-mer template/primer n (G∙C match), 15-mer template/primer n + 1 (G∙C match), 15-mer template/primer n (G~G mismatch), or 15-mer template/primer n + 1 (G~G mismatch). ^3^ *K*_d_ denotes the dissociation constant for strand dissociation (∆*G*^0^_310_ = −*RT*ln*K*_d_).

## Supplementary Materials

Supplementary material can be found at https://www.mdpi.com/1422-0067/21/20/7806/s1. Figure S1. Inhibition of RNA synthesis by SP6 and T7 RNA polymerases on the pSP73KB plasmid modified by AMD or cisplatin; Figure S2. Steady-state kinetics of dNTP incorporation by KF^−^ and pol η opposite G unplatinated or platinated by AMD; Figure S3. Steady-state kinetic analysis—graphical representation of individual dNTP insertions by KF^−^; Figure S4. Steady-state kinetic analysis—graphical representation of individual dNTP insertions by polymerase η; Figure S5. Exemplary MST time traces used to determine *K*_d_ at 298 K (n − 1, n, n_G_, n_G_ + 1) and 310 K (n + 1) for sets of the templates (undamaged and containing AMD adduct) hybridized with 4 nM primers (n − 1, n, n + 1) or 1 nM mismatched primers (n_G_ and n_G_ + 1).

## Abbreviations

ACR	[PtCl(en)(L)](NO_3_)_2_ (en = ethane-1,2-diamine, L = 1-[2-(acridin-9-ylamino)ethyl]-1,3-dimethylthiourea)
AMD	[PtCl(en)(L)](NO_3_)_2_ (en = ethane-1,2-diamine, L = *N*-[2-(acridin-9-ylamino)ethyl]-*N*-methylpropionamidine)
TLS pol	translesion DNA polymerase
DNA pol	DNA polymerase
KF^−^	Klenow fragment of DNA polymerase I (the exonuclease deficient)
Pol η	DNA polymerase η
MST	microscale thermophoresis
TLS	translesion DNA synthesis
*r*_b_	number of molecules of the platinum complex bound per nucleotide residue
dien	diethylenetriamine (1,4,7-triazaheptane)
CT	calf thymus
dNTP	2′-deoxyribonucleotide-5‘-triphosphate
PAA	polyacrylamide
Cy5	far-red fluorescent label for protein or nucleic acid conjugates
*K*_d_	equilibrium dissociation constant
RE	relative efficiency
DTT	dithiothreitol
BSA	bovine serum albumin
