# Supplementary material for: Thermodynamic Insights by Microscale Thermophoresis into Translesion DNA Synthesis Catalyzed by DNA Polymerases Across a Lesion of Antitumor Platinum–Acridine Complex
Source: Int J Mol Sci. 2020 Oct 21;21(20):7806. doi: 10.3390/ijms21207806 (PMC7589001; doi:10.3390/ijms21207806)
Supplement: Supplementary file 1 [file ijms-21-07806-s001.pdf]

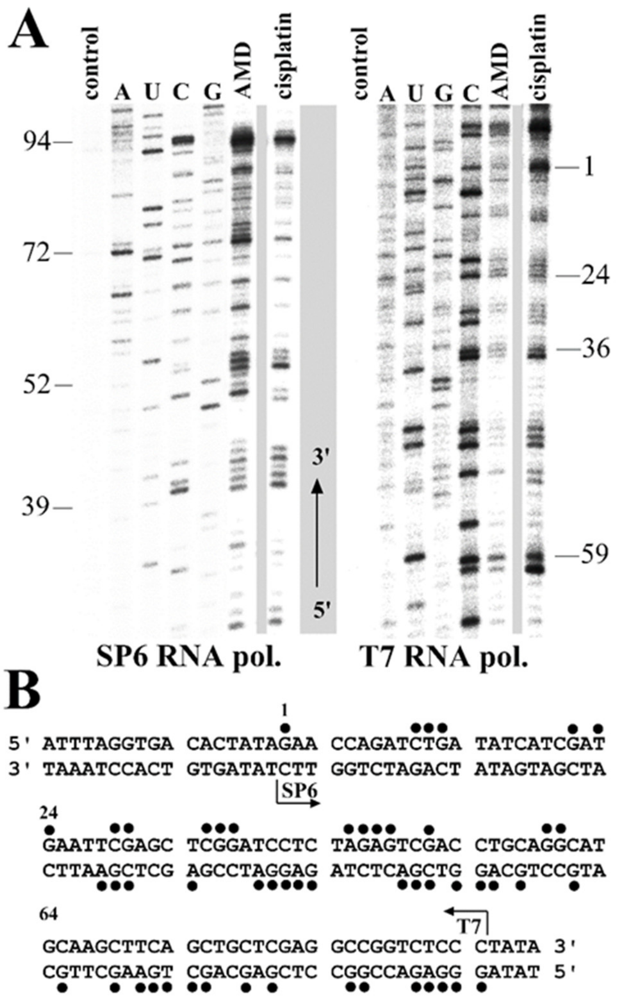

Figure S1. Inhibition of RNA synthesis by SP6 and T7 RNA polymerases on the pSP73KB plasmid modified by AMD or cisplatin. (**A**) Autoradiograms of 8% polyacrylamide/8 M urea sequencing gels showing inhibition of RNA synthesis by SP6 (left) or T7 RNA polymerases (right) on the plasmid DNA containing adducts of platinum complexes. Lanes: control, unmodified template; A, U, C, and G, chain terminated marker RNAs; AMD and cisplatin, the template modified by AMD at $r_b = 0.02$ (SP6 RNA pol.) or $r_b = 0.01$ (T7 RNA pol.), or cisplatin at $r_b = 0.01$, respectively. (**B**) Schematic diagram showing the portion of the sequence used to monitor inhibition of RNA synthesis by platinum complexes. The arrows indicate the start of the SP6 and T7 RNA polymerase, which used as a template the bottom or upper strand of the pSP73KB DNA, respectively. (●) major stop signals for DNA modified by AMD. The numbers correspond to the nucleotide numbering in the sequence map of the pSP73KB plasmid.

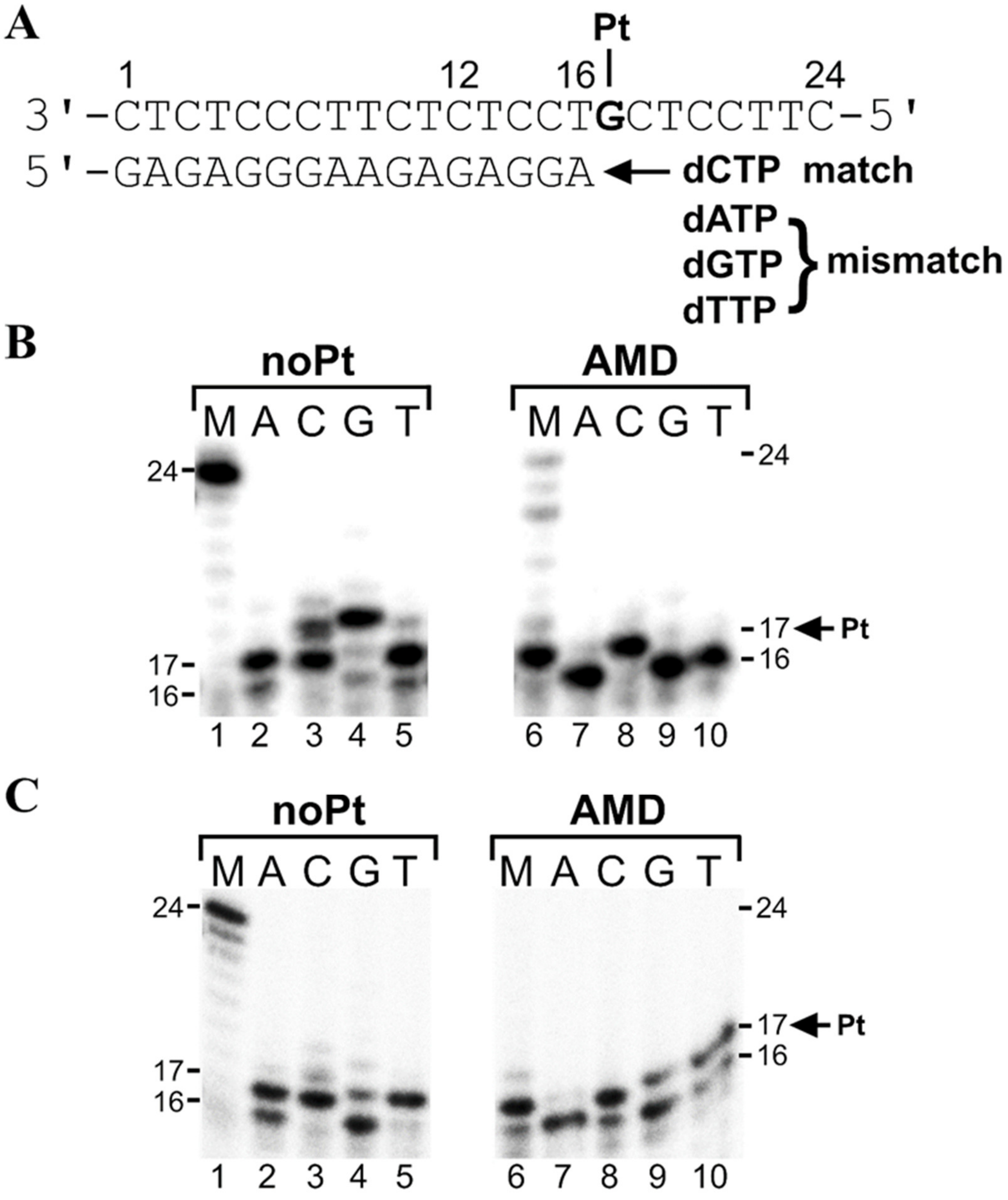

Figure S2. Steady-state kinetics of dNTP incorporation by KF$^-$ and polη opposite **G** unplatinated or platinated by AMD. (**A**) The nucleotide sequences of the template and the primer, and offered dNTPs. Representative images of the products of KF$^-$ (**B**) and polη (**C**) reactions resolved on 15% polyacrylamide gels and visualized by radiography. The capacity to elongate a 5′-^{32}P-labeled 16mer primer annealed to the unmodified 24mer template (panels B and C, lanes 1–5, „noPt“) or the 24mer template containing AMD, lanes 6–10, „AMD“, respectively, in the presence of the mixture of all four deoxyribonucleotides 5′-triphosphates (lanes M) or complementary dCTP or non-complementary nucleotides individually (the corresponding nucleotides are marked by letters A, C, G, T above the gel) at 298 K (KF$^-$) or 310 K (polη) for 60 min. The pause sites (the product lengths) are shown on the right or left sides of the gels.

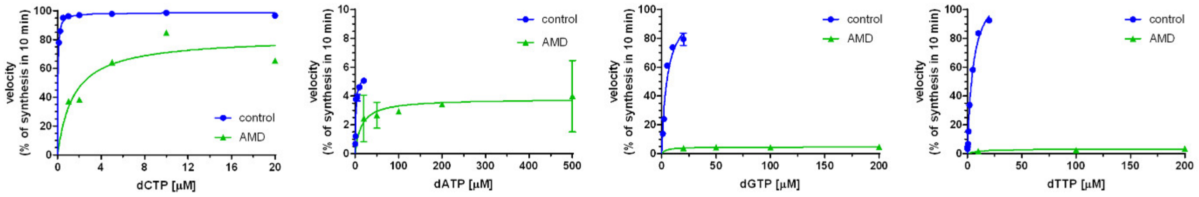

Figure S3. Steady-state kinetic analysis - graphical representation of individual dNTP insertions by KF^{-}. Data are means (±SEM) from at least two different experiments, for some points, the error bar is shorter than the height of the symbol. Fitting was to a hyperbolic equation in GraphPad Prism v. 7.04, and V_{max}, t, and K_m values are presented in Table 1.

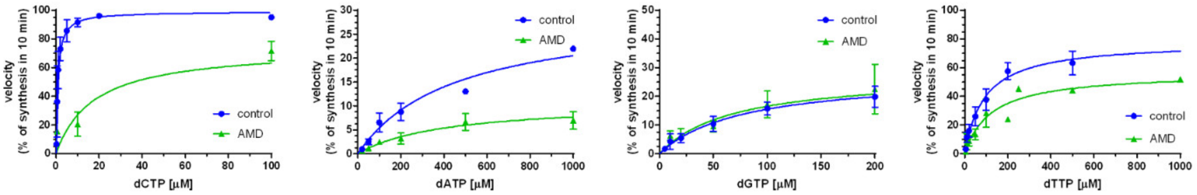

Figure S4. Steady-state kinetic analysis - graphical representation of individual dNTP insertions by polymerase η**.** Data are means (±SEM) from at least two different experiments; for some points, the error bar is shorter than the height of the symbol. Fitting was to a hyperbolic equation in GraphPad Prism v. 7.04, and V_{max}, t, and K_m values are presented in Table 2.

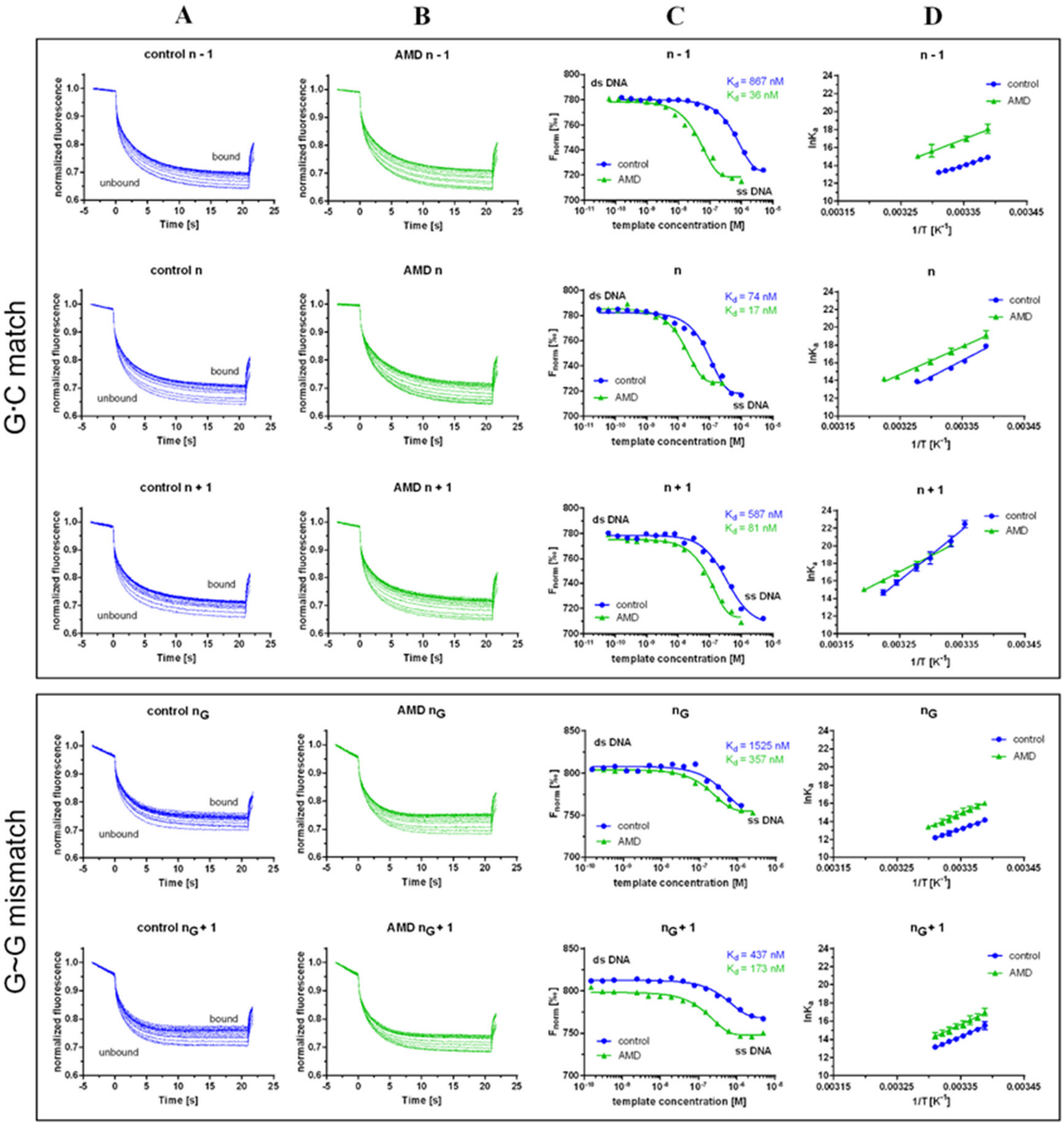

Figure S5. Exemplary MST time traces used to determine K_ds at 298 K (n - 1, n, n_G, n_G + 1) and 310 K (n + 1) for sets of the templates (undamaged and containing AMD adduct) hybridized with 4 nM primers n - 1, n, n + 1 or 1 nM mismatched primers n_G and n_G + 1. MST timetraces corresponding to the titration of the template strand against Cy5-labeled primer are shown in columns A and B: Blue lines, undamaged templates; green lines, templates containing AMD adduct, all normalized to a starting value of 1.0. Plots in the column C represent the change in MST signals that was fitted to yield K_d values. Single-stranded (ss) DNA yields a stronger MST response than double-stranded (ds) DNA. Column D represents van't Hoff plots of the n - 1, n, n + 1 or mismatched n_G and n_G + 1 primer-template hybridization reactions. K_d values were determined for each temperature by fitting the T-Jump or thermophoresis signal and plotted as ln(K_a = 1/K_d) vs. 1/T (K). ΔH was obtained from the slope m of the linear fit as m = $-H^o/R$. Under the assumption that ΔH is constant in the relatively small linear range of the van't Hoff plot, ΔS was directly derived from the plot as y(0) = $\Delta S^o/R$. The universal gas constant R = 8.314 J $K^{-1}mol^{-1}$. Data are means (±SD) from at least two different experiments; coefficient of determination $r^2 \geq 0.98$. Thermodynamic parameters are presented in Table 3.
